# The failure of routine rapid HIV testing: a case study of improving low sensitivity in the field

**DOI:** 10.1186/1472-6963-10-73

**Published:** 2010-03-22

**Authors:** Benjamin J Wolpaw, Catherine Mathews, Mickey Chopra, Diana Hardie, Virginia de Azevedo, Karen Jennings, Mark N Lurie

**Affiliations:** 1 Health Systems Research Unit, South African Medical Research Council, Cape Town, South Africa; 2 School of Public Health and Family Medicine, University of Public Health, Cape Town, South Africa; 3 School of Public Health, University of the Western Cape, Bellville, South Africa; 4 National Health Laboratory Service and Division of Virology, University of Cape Town, Cape Town, South Africa; 5 Cape Town City Health Department, City of Cape Town, Cape Town, South Africa; 6 Department of Community Health, Brown University Medical School, Providence, USA

## Abstract

**Background:**

The rapid HIV antibody test is the diagnostic tool of choice in low and middle-income countries. Previous evidence suggests that rapid HIV diagnostic tests may underperform in the field, failing to detect a substantial number of infections. A research study inadvertently discovered that a clinic rapid HIV testing process was failing to detect cases of established (high antibody titer) infection, exhibiting an estimated 68.7% sensitivity (95% CI [41.3%-89.0%]) over the course of the first three weeks of observation. The setting is a public service clinic that provides STI diagnosis and treatment in an impoverished, peri-urban community outside of Cape Town, South Africa.

**Methods:**

The researchers and local health administrators collaborated to investigate the cause of the poor test performance and make necessary corrections. The clinic changed the brand of rapid test being used and later introduced quality improvement measures. Observations were made of the clinic staff as they administered rapid HIV tests to real patients. Estimated testing sensitivity was calculated as the number of rapid HIV test positive individuals detected by the clinic divided by this number plus the number of PCR positive, highly reactive 3^rd ^generation ELISA patients identified among those who were rapid test negative at the clinic.

**Results:**

In the period of five months after the clinic made the switch of rapid HIV tests, estimated sensitivity improved to 93.5% (95% CI [86.5%-97.6%]), during which time observations of counselors administering tests at the clinic found poor adherence to the recommended testing protocol. Quality improvement measures were implemented and estimated sensitivity rose to 95.1% (95% CI [83.5%-99.4%]) during the final two months of full observation.

**Conclusions:**

Poor testing procedure in the field can lead to exceedingly low levels of rapid HIV test sensitivity, making it imperative that stringent quality control measures are implemented where they do not already exist. Certain brands of rapid-testing kits may perform better than others when faced with sub-optimal use.

## Background

The availability in low and middle income countries of effective medication for HIV in the form of highly active antiretroviral therapy has created the need for efficient and accurate HIV testing programs to identify candidates for treatment [1]. HIV testing also remains critical to prevention efforts as well as programs providing psychological support. The rapid HIV antibody test, performed on whole blood collected from a finger prick, is the diagnostic tool of choice due to low cost, relative ease of use, and speed in obtaining results [2]. Current WHO guidelines for the use of rapid HIV testing in resource constrained settings recommend the use of rapid-test kits with >99% sensitivity and there are a number of available options with this level of accuracy [1].

There is evidence, however, to suggest that rapid test kits may underperform in the field, failing to detect a substantial number of infections. A recent study from KwaZulu-Natal, South Africa found that the sensitivity of four different rapid tests kits when administered by nurses at antenatal sites was 92.5-97.3%, and 100% when performed by laboratory technicians (compared with ELISA gold standard) [3]. A 2008 South African government report, commissioned in response to descriptions of unreliable rapid test results in local healthcare facilities, found that weakly positive results could be difficult to correctly interpret and might lead to false negative diagnoses in the field. Yet rapid test sensitivity in the field can be excellent when properly monitored: in four African countries that perform routine laboratory retesting of a portion of field results sensitivity has ranged from 98.1% to 99.7% [4].

WHO has acknowledged the need for ongoing quality control and assessment measures in the guidelines for country-based implementation of rapid HIV testing. These guidelines recommend competency assessments of personnel who perform the tests, site visits to observe testing, and external quality assurance based on retesting a proportion of specimens by reference laboratories [5]. Yet there is a widely held assumption among health administrators that the user-friendly rapid tests are as reliable and accurate in the field as in the laboratory, requiring little monitoring to ensure the quality of results. A 2007 review of eleven African countries with rapid testing programs found that only seven had a system involving laboratory retesting, four conducted periodic on site observations, and two utilized proficiency panel testing [4].

We report on the poor sensitivity of rapid HIV tests in the field and the contrasting performance of different testing kits. It is our hope that this account will challenge the assumption that quality control around HIV testing need not be a high priority, lending urgency and perspective to ongoing efforts to establish quality control measures in resource constrained settings.

## Methods

This report is from a public service clinic in an informal settlement area of Cape Town, South Africa. Unemployment there is high and many people live in abject poverty: 25% of households report having no income and over 50% of houses are informal dwellings or shacks [6,7]. Antenatal HIV prevalence was 32.6% for the district in 2005. There are significant barriers to the optimal delivery of HIV testing services at clinics including high staff attrition rates, insufficient in-service training for new staff, and large numbers of patients.

From August 2008 through March 2009, the MRC AHI study (approved by the Ethics Review Committee of University of Cape Town) performed surveillance for acute stage HIV infection among patients testing for HIV at the clinic. The clinic routinely offers voluntary HIV testing using a serial testing algorithm, whereby one rapid test is administered and a second test is used for confirmation of a positive result.

Adults (≥ 18 yrs) who were rapid antibody test negative or inconclusive during routine clinic testing were referred to the study. Following informed consent, blood was taken for further testing using HIV-1 DNA PCR (Roche Amplicor). PCR positive samples were then tested with a 3^rd ^generation HIV ELISA (Dade Behring) to quantitatively evaluate antibody levels.

Patients who were PCR positive with zero to moderate ELISA reactivity were classified as having acute HIV infection, which describes the first weeks to months of infection. Rapid tests may fail to detect individuals during this time owing to low levels of HIV antibody [8]. Soon after beginning the study, however, we unexpectedly discovered patients referred to us by the clinic health workers with negative rapid test results who were PCR positive and strongly ELISA reactive for HIV. These individuals had established (non-acute) HIV infections and should have been detected 99-100% of the time by rapid testing in the clinic, according to the rapid test package insert. Retesting of the blood samples in the laboratory with rapid tests yielded positive results.

A 2008 South African government report, commissioned in response to descriptions of unreliable rapid test results in local healthcare facilities, found that weakly positive rapid test results could be difficult to correctly interpret and might lead to false negative diagnoses in the field. For this reason, a change in the test brand had already been planned. This change occurred three weeks following the beginning of our observations. The names of the two tests are withheld for the purpose of this report. It is the second of these that was used for all laboratory retesting with rapid tests as explained above.

During the month following the change in rapid tests, two clinic testing personnel were evaluated for adherence to proper protocol (as specified in package inserts) while performing rapid testing five times each on real patients. The regional manager of the clinic subsequently ordered additional testing quality improvement measures for all area clinics, which occurred five months after the change in rapid tests. These measures consisted of retraining of staff in the use of rapid HIV tests, distribution of an illustrated guide with clear instructions for test use, and the introduction of electronic timers to accurately measure when results should be read.

We are reporting on the clinic's sequential use of two different brands of rapid test kits and over a period before and after quality improvement measures. By detecting cases of established HIV infection among those diagnosed as HIV negative by the routine clinic testing procedure, we were able to estimate testing sensitivity for different time periods. Estimated testing sensitivity was calculated as the number of rapid HIV test positive individuals detected by the clinic divided by this number plus the number of patients with established infections identified among those who were rapid test negative at the clinic. Only those clinic patients who enrolled in the research study were tested as part of our study. Thus, it is probable that cases of established HIV infection went undetected, making our observed sensitivities high estimates.

## Results

In the period preceding the switch of rapid test kits, estimated sensitivity was 68.7% (see table 1). After the clinic began to use the new rapid test kits, testing sensitivity appears to have improved. Observed sensitivity was 93.5% over five months. At this point retraining of staff and the introduction of quality improvement measures occurred and observed sensitivity improved slightly to 95.1% over the final two months of observation.

**Table 1:** Estimated rapid-test sensitivity during three periods of observation

Test Used at Clinic	Total testing at clinic	Total Testing Positive at Clinic	Eligible Participants Enrolled in Study (%)	Established HIV infections detected by PCR/ELISA	**Observed Testing Sensitivity (95% CI)**
Test Brand 1	69	11	82.7%	5	**68.7% (41.3%-89.0%)**

Test Brand 2 (before quality improvement measures)	759	87	65.9%	6	**93.5% (86.5%-97.6%)**

Test Brand 2 (following quality improvement measures)	349	39	80.2%	2	**95.1% (83.5%-99.4%)**

Conditions of storage were found to be adequate throughout the entire period of the study. Observations shortly after the change in rapid tests identified several problems that could conceivably result in a false negative result:

*Technique - Use of a capillary tube to apply blood to the kits and proper administration of the chase buffer was inconsistent;

*Time for reading of results - The package insert of the rapid tests in use states that at least 15 minutes be allowed to pass between the application of patient blood and the reading of results. This only occurred in 1 of 10 observed cases and the waiting time was above 10 minutes in only 3 cases.

## Discussion

We observed a period during which rapid HIV testing sensitivity at a clinic offering routine-testing services was estimated at 68.7% (95% CI [41.3%-89.0%]). At a later date, observations were made of poor testing practices on the part of clinic staff. Because staff remained the same during this time period we hypothesize that poor adherence to protocol explains the low test sensitivity.

After the change in rapid test kits, and before any re-training or quality improvement measures were implemented, there was a sizeable increase in observed testing sensitivity. This suggests there is variation among tests in accuracy under conditions of suboptimal use. In overburdened and under resourced settings, rapid tests will not always be administered in perfect accordance with recommended protocol. Future research should compare different rapid test kits as used in the field and determine the true operating characteristics of these tools.

The sample size in this study for the use of the first test kit is small because of the short time period between the beginning of observation and the change of rapid tests. This study was not originally designed to compare rapid test kits and we can make no recommendations regarding the use of one test kit compared with another.

## Conclusions

In 2008, over 9,000 individuals were diagnosed with HIV in clinics in the community from which we are reporting. If testing sensitivity during this time was the high end of the 95% CI of our observations before any changes were made (89.0%), more than 1,100 additional HIV positive individuals received negative results. These people have lost the ability care properly for themselves, access treatment, and protect their sexual partners.

The chance detection of poor test performance in the clinic where we were working allowed steps to be taken which led to subsequent improvements in sensitivity. In the context of other evidence of poor rapid test performance in the field, our findings support an urgent call for the implementation of stringent quality control and assurance measures where they do not already exist. These measures should include routine laboratory retesting of a random sample of rapid test results and evaluation of adherence to testing protocol on the part of field staff.

## Abbreviations

AHI: Acute HIV Infection; ELISA: Enzyme Linked Immunosorbent Assay; MRC: Medical Research Council; PCR: Polymerase Chain Reaction; STI: Sexually Transmitted Infection; WHO: World Health Organization.

## Competing interests

Two of the authors of this manuscript are employed by the City of Cape Town Health Department, the governmental organization responsible for the clinic in question. The authors have no other conflicts of interest to declare.

## Authors' contributions

BW was primarily responsible for the planning, implementation, and management of the project; he was the primary author of this manuscript. CM was heavily involved with the planning, implementation, and management of the research project and assisted in the writing of this manuscript. MC was involved with the development of the research project in question, participated in key decision-making processes, and assisted with the writing of this manuscript. DH was responsible for all laboratory aspects of the study; she advised with the choice of tests used and oversaw the administration of these tests and interpretation of results. VA is the City of Cape Town Health Department sub-district manager responsible for the clinic in question; she was responsible for the changes made in the clinic during the course of this study and assisted with the writing of this manuscript. KJ is the head of AIDS/STI/TB services for the City of Cape Town Health Department and was also responsible for changes made and assisted with the writing of this manuscript. ML was involved with the planning of the research project in question and the preparation of this manuscript. All authors have read and approved the final manuscript.

## Pre-publication history

The pre-publication history for this paper can be accessed here:

http://www.biomedcentral.com/1472-6963/10/73/prepub
